# [EMIM][Tf2N]-Modified Silica as Filler in Mixed Matrix Membrane for Carbon Dioxide Separation

**DOI:** 10.3390/membranes11050371

**Published:** 2021-05-19

**Authors:** Siti Nur Alwani Shafie, Nik Abdul Hadi Md Nordin, Muhammad Roil Bilad, Nurasyikin Misdan, Norazlianie Sazali, Zulfan Adi Putra, Mohd Dzul Hakim Wirzal, Alamin Idris, Juhana Jaafar, Zakaria Man

**Affiliations:** 1 Department of Chemical Engineering, Universiti Teknologi PETRONAS (UTP), Seri Iskandar 32610, Malaysia; alwanishafie93@gmail.com (S.N.A.S.); mdzulhakim.wirzal@utp.edu.my (M.D.H.W.); zakaman@utp.edu.my (Z.M.); 2 Faculty of Applied Science and Enginering, Universitas Pendidikan Mandalika UNDIKMA, Jl. Pemuda No. 59A, Mataram 83126, Indonesia; 3 Faculty of Engineering Technology, Universiti Tun Hussein Onn Malaysia (UTHM), Parit Raja 86400, Malaysia; nurasyikin@uthm.edu.my; 4 Centre of Excellence for Advanced Research in Fluid Flow (CARIFF), Faculty of Mechanical Engineering, Universiti Malaysia Pahang (UMP), Pekan 26600, Malaysia; azlianie@ump.edu.my; 5 PETRONAS Group Technical Solutions, Project Delivery and Technology, PETRONAS, Kuala Lumpur 50050, Malaysia; zulfan.adiputra@petronas.com.my; 6 Department of Engineering and Chemical Sciences, Karlstad University, SE-65188 Karlstad, Sweden; Alamin.Abdulgadir@kau.se; 7 Advanced Membrane Technology Research Centre (AMTEC), Universiti Teknologi Malaysia (UTM), Skudai 81310, Malaysia; juhana@petroleum.utm.my

**Keywords:** CO_2_ separation, mixed matrix membrane, silica, [EMIM][TF_2_N], ionic liquid

## Abstract

This study focuses on the effect of modified silica fillers by [EMIN][Tf_2_N] via physical adsorption on the CO_2_ separation performance of a mixed matrix membrane (MMM). The IL-modified silica was successfully synthesized as the presence of fluorine element was observed in both Fourier transform infrared spectroscopy (FTIR) and X-ray photoelectron spectrometer (XPS) analyses. The prepared MMMs with different loadings of the IL-modified silica were then compared with an unmodified silica counterpart and neat membrane. The morphology of IL-modified MMMs was observed to have insignificant changes, while polymer chains of were found to be slightly more flexible compared to their counterpart. At 2 bar of operating pressure, a significant increase in performance was observed with the incorporation of 3 wt% Sil-IL fillers compared to that of pure polycarbonate (PC). The permeability increased from 353 to 1151 Barrer while the CO_2_/CH_4_ selectivity increased from 20 to 76. The aforementioned increment also exceeded the Robeson upper bound. This indicates that the incorporation of fillers surface-modified with ionic liquid in an organic membrane is worth exploring for CO_2_ separation.

## 1. Introduction

Membranes for gas separation have been commercially attractive to separate carbon dioxide (CO_2_) from methane (CH_4_) compared to other conventional methods (i.e., absorption, adsorption) due to the ease of scale-up, simple process design and minimal energy requirements. Over recent decades, efforts have been made to improve the separation factor by incorporating inorganic materials, known as mixed matrix membranes (MMMs). Porous (i.e., zeolite [1], activated carbon [2], porous silica and metal–organic framework [3,4]) and non-porous (i.e., graphene oxide, clay mineral [5] and non-porous silica) inorganic particles have been reported to improve physicochemical properties and gas separation performance [6,7,8]. 

Silica particles (Sil) as inorganic fillers are attractive with superior properties such as selectivity, rigidity, ease of processing, large surface area and chemical and thermal stability with well-defined and controllable morphology and porosity [9]. Moreover, silica particle surfaces can be easily modified to alter their specific properties. The incorporation of silica particles in a polymer matrix could enhance the separation performance by disruption in the polymer chain, resulting in large free volumes [10]. Ahn et al. reported that using fumed silica as a filler increases the total free volume of the polymer phase, creating more diffusion pathways for gas diffusion and improved CO2 permeability from 6.3 to 19.7 Barrer at 20% of silica content [11]. It should, however, be noted that incorporating silica in a polymer matrix often leads to interfacial defects [12,13,14]. This phenomenon is observed as unselective void formation, often leading to deterioration of the membrane’s gas pair selectivity.

Moreover, filler surface modification is often implemented to improve the adhesion between organic/inorganic materials. It is typically achieved using a modification agent that is compatible with polymer phases that chemically react with the filler and act as the bridges at the interphase [15], and silane is among the most common modification agent employed for MMMs [16,17,18,19]. Zhang et al. [19] reported that the presence of 3-aminopropyltriethoxysilane (APTS) in graphene oxides (GOs) improves the compatibility between the filler and polymer phase (PEBAX^®^ 1657), eliminating the presence of interfacial defects. In addition, with the absence of interfacial defect, the presence of CO_2_-philic groups (amino) in the APTS resulted in higher affinity towards CO_2_, thus simultaneous increases in CO_2_ permeability and CO_2_/CH_4_ selectivity were reported.

Recently, ionic liquid (IL) has emerged as an alternative to overcome the polymer–filler interfacial defects and improve CO_2_ affinity [20,21]. The tenability of IL properties allows IL to act as a bridge between polymer and filler. Specifically, imidazole-based IL could enter into filler pores while forming specific interactions with the polymer matrices (i.e., dipole–dipole interaction and hydrogen bond) [22]. For example, Ahmad et al. [23] reported that impregnated zeolite SAPO-34 by IL (through immersion) minimized the interfacial defects when incorporated into polysulfone (PSF) matrices. The CO_2_ separation performance of PSF/SAPO-34/IL shows that CO_2_ permeance increased by 11% while CO_2_/CH_4_ selectivity increased by 456% compared to a PSF/SAPO-34 membrane.

It should be noted that the previously mentioned method is only applicable for porous fillers. However, the impregnation of IL could eliminate the molecular sieving effect caused by the filler’s pores and the improved separation would only be contributed to by the increase in the solubility of gases. Therefore, it can be postulated that modification using IL on non-porous filler would also result in similar improved separation. In this work, the effects of IL-modified Sil loading on membrane properties and CO_2_ separation performance are investigated. MMMs using unmodified Sil particles with different loadings are also prepared for comparison. 

## 2. Materials and Methods

### 2.1. Materials

CALIBRE™ polycarbonate (PC) was purchased from Trinseo ( Berwyn, PA, USA); dicholoromethane (DCM), tetraethyl orthoailicate (TEOS), hydrochloric acid (HCl, 37%), and ethanol (EtOH) (99.5% purity) were purchased from Merck (Darmstadt, Germany), and ionic liquid 1-Ethyl-3-methylimidazolium bis(trifluoromethylsulfonyl)imide ([EMIM][TF_2_N]) was procured from Sigma-Aldrich (St. Louis, MO, USA). All chemicals were used without further purification.

### 2.2. Silica Particle Synthesis

Silica particles were synthesized using the standard sol–gel process adapted from the literature [24]. TEOS (30 mL) and EtOH (31 mL) were first added to a round bottom flask with a reflux condenser and the solution was stirred for 5 min. Diluted HCl solution (0.01 mL of HCl and 38 mL of distilled water) was then added into the flask and further stirred and heated at 60 °C for 90 min. The sol–gel solution was then cooled to room temperature before drying in an oven at 60 °C overnight. The collected silica was washed with distilled water and ethanol before being filtered using a vacuum pump. The silica particles were dried in a vacuum oven at 105 °C overnight to ensure the complete removal of the solvent.

### 2.3. IL-Modified Silica Preparation

IL-modified silica particles were prepared based on the physical adsorption of [EMIM][Tf_2_N] as IL on the surface of silica particles. Silica particles were added into a beaker with 10.0 g of ethanol and [EMIM][TF_2_N] (10.0 mL). The reaction mixture was stirred at room temperature, filtered for 12 h, washed with ethanol, filtered using vacuum filtration, and dried in an oven at 60 °C to ensure the complete removal of solvent. 

### 2.4. Membrane Preparation

All developed membranes were prepared based on PC (20 wt%) and DCM (80wt%). Amounts of 1 wt%, 2 wt%, and 3 wt% (based on the weight of total solid) of filler (unmodified and modified silica) were used. Briefly, a predetermined amount of filler (Table 1) was dispersed in the DCM and sonicated for 5 min. Then, 20% of total polymer was initially added and stirred until dissolved for priming. The remaining polymer was then added and stirred at room temperature until a homogeneous solution was obtained. The dope solution was then degassed at room temperature for 3 h using an ultrasonicator to remove the bubbles formed during the mixing process. The membrane solution was then hand casted on a glass plate with a thickness of approximately 100 µm using a casting knife. The membrane sheet was dried at room temperature for 24 h to ensure complete evaporation of the solvent. The pristine PC and PC-Sil membranes were prepared using the same process without the filler and pristine silica particles as filler, respectively, based on compositions tabulated in Table 1. 

### 2.5. Characterization 

Fourier transform infrared spectrometry (FTIR) analysis was carried out to determine the functional group change of silica powder after blending with [EMIM][TF_2_N]. FTIR analysis was carried out using a Perkin-Elmer Spectrum 1 Fourier transform infrared spectrometer (Lambda 365, Waltham, MA, USA) and Spectra One software. The powder sample and KBr were ground before mixing. The powder was then added into a die-set to form a pellet before being placed in the sample holder. The spectrum was studied by co-addition of 20 scans in the range of 400–4000 cm^−1^. Within this range, the organic component converted the radiation into vibration. 

X-ray photoelectron spectroscopy (XPS) analysis was conducted to measure the elemental composition at the parts per thousand range, empirical formula, chemical state, and electronic state of the elements that exist within the material. XPS analysis was conducted to determine the Si, O, C, F, N elements in the silica particles and the modified silica particles. 

The structures of the morphology of fabricated flat sheet membrane were analyzed using field emission scanning electron microscopy (FESEM, Zeiss Supra55, Zeiss, Oberkochen, Germany). It was used to determine the structural assessment of both the surface and cross-section of the membranes. This analysis was conducted to check the morphological criteria. In order to obtain smooth and clean cross-section images, the membranes were immersed in liquid nitrogen for a few seconds and coated in gold/platinum. The samples were mounted horizontally and vertically on a circular stainless steel plate holder in order to obtain both surface and cross-section view monographs. All membranes were analyzed in a magnification range of 5 k to 1000 k using 15 kV accelerating voltage. 

Thermal gravimetric analysis (TGA) was carried out to estimate the thermal stability, material composition, purity, and the amount of remaining solvent left in the membranes after drying. This thermal analysis was conducted to determine the ideal temperature the membrane can withstand. For this analysis, the membranes were thermally characterized by a thermal gravimetric analyzer (TGA 4000, Perkin Elmer, Waltham, MA, USA) in which the samples were heated at temperatures ranging from 25 °C to 800 °C at a 10 °C/min heating rate with inert nitrogen (N_2_). 

Differential scanning calorimetry (DSC) was used to determine the glass transition temperature of the membrane (T_g_). In this study, DSC was carried out to analyze the effect of silica and IL-modified silica in the polymeric membrane on the membrane glass transition temperature. Firstly, small and clean membranes were placed on the DSC pans. Samples were heated from 30 °C to 800 °C at a rate of 10 °C /min in N_2_ conditions. However, after reaching 250 °C, the samples were naturally cooled to remove the thermal history. Under the same procedure, a second scan was conducted. To get the T_g_ value of membranes, the second scan values were used. 

X-ray diffraction (XRD) was conducted for structural characterization of pure PC, PC-Sil MMMs, and PC-Sil-IL MMMs. The setup (XRD, D8 Advance, Bruker, Billerica, MA, USA) generated spectra of XRD scans of scattering intensities in counts per second as a function of the diffraction angle. The samples were run in a scan range of 10° to 80°. The intersegmental distance or *d*-spacing of the membrane was calculated using Bragg’s law equation:

(1) nλ=2d⋅sinθ

where *n* is the order of reflection, *λ* is the wavelength of CuKα (1.54 Å), and *d* is the interplanar spacing. 

### 2.6. Gas Permeation Test

The permeation experimentation for pure CO_2_ and CH_4_ gas was conducted using a membrane gas permeation testing unit with 2–10 bar feed pressure at room temperature (25 °C). The membranes were cut into 58 mm diameter circles using a circle cutter and were put into the permeation cell [25]. The flow rate of the gas permeate was measured using a bubble flow meter (Humonics 420) and the reading was repeated three times. The permeability, *P* (unit barrer), was then evaluated using the following equation:

(2) $${\frac{P}{l}}_{}=\frac{Q_{STP}{}_{}}{A\cdot \Delta P}$$

(3) $$P=\frac{Q_{STP}\cdot l}{A\cdot \Delta P}$$

where *Q_STP_* is the permeate flow rate at standard temperature and pressure, *A* is the effective surface area of the membrane film, ∆*P* is the pressure gradient across the membrane. The unit of gas permeability is expressed in Barrer (Equation (4)):

(4) $$\mathrm{Barrer}=10^{-10}.\frac{{\mathrm{cm}}^{3}(\mathrm{STP})\cdot \mathrm{cm}}{s\cdot {\mathrm{cm}}^{2}\cdot \mathrm{cmHg}}$$

The gas pair CO_2_/CH_4_ selectivity of a membrane was evaluated by the ratio of the permeability of more permeable gas over the less permeable gas (CO_2_/CH_4_). The separation factor can be found as follows:

(5) αAB=PAPB

## 3. Results

### 3.1. Silica and Silica-IL Characterization

**Figure 1:**
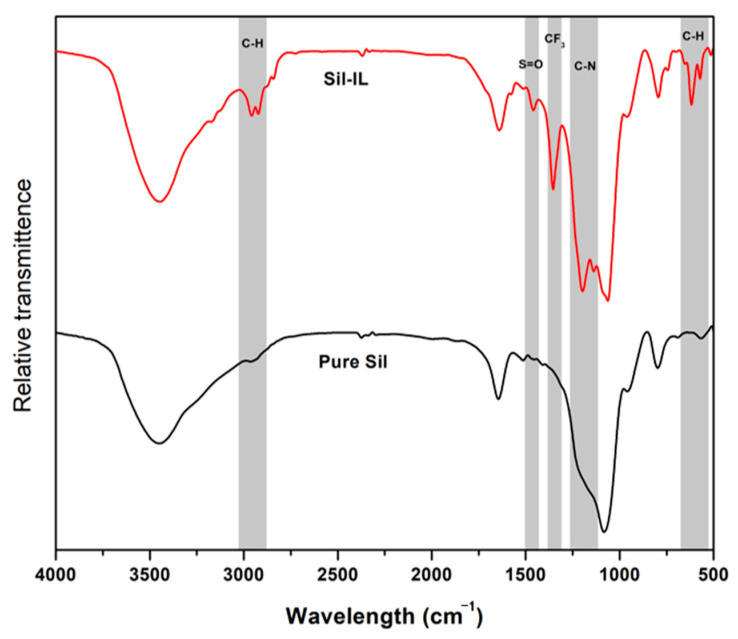
FTIR spectra for pure silica and Sil-IL particles.

The FTIR analyses of prepared pure silica and Sil-IL are presented in Figure 1. For pure silica, the 3500 cm^−1^ peak shows the hydroxyl group O-H bond while that of 1100–1000 cm^−1^ shows the Si-O-Si stretching, which are similar to those reported in the literature [26], thus confirming the successful synthesis of the silica particles. Upon modification with [EMIM][Tf_2_N], a few additional peaks were observed. A peak at a wavelength of 2800 cm^−1^ represents the C-H stretching and that at 700 cm^−1^ represents the C-H wag from the imidazolium ring [27]. The 1300 cm^−1^ peak shows that the S=O bond originated from the anion side of the ionic liquid. At the 1200 cm^−1^ peak, the CF_3_ characteristic peak from [Tf_2_N] is also present, in accordance with the literature [28]. It is also noticed that a C-N peak is observed at 1200 cm^−1^ [29]. Thus, it can be safely confirmed that IL has been successfully attached onto the surface of the silica particles.

The elemental composition of the silica and modified silica particles is tabulated in Table 2. For pure silica particles, silicon and oxygen elements were observed, further confirming that the particles were successfully synthesized. When surface modified with [EMIM][TF_2_N], it can be observed that additional fluorine, nitrogen, and carbon elements were present while the oxygen element decreased. This suggests that IL replaces the oxygen element in the silica particles, thus confirming the successful modification [30]. 

**Figure 2:**
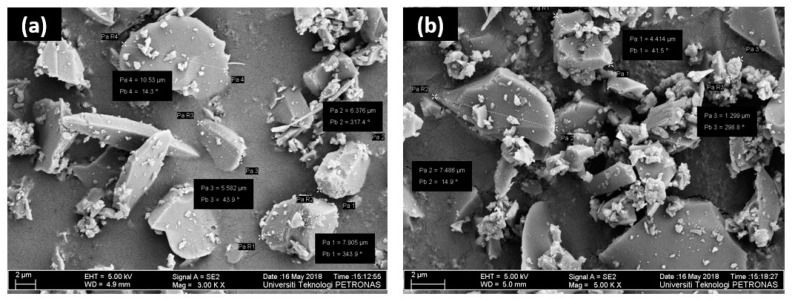
FESEM image of (**a**) pure Sil particles (**b**) IL-modified Sil particles.

Figure 2 shows the morphology of the synthesized Sil and Sil-IL particles. It can be observed that the Sil particles have an irregular shape, with sizes varying from 2–10 µm. Upon modification with [EMIM][TF_2_N], there was no prominent difference in the morphology of the particles. 

### 3.2. Membrane Characterization

**Figure 3:**
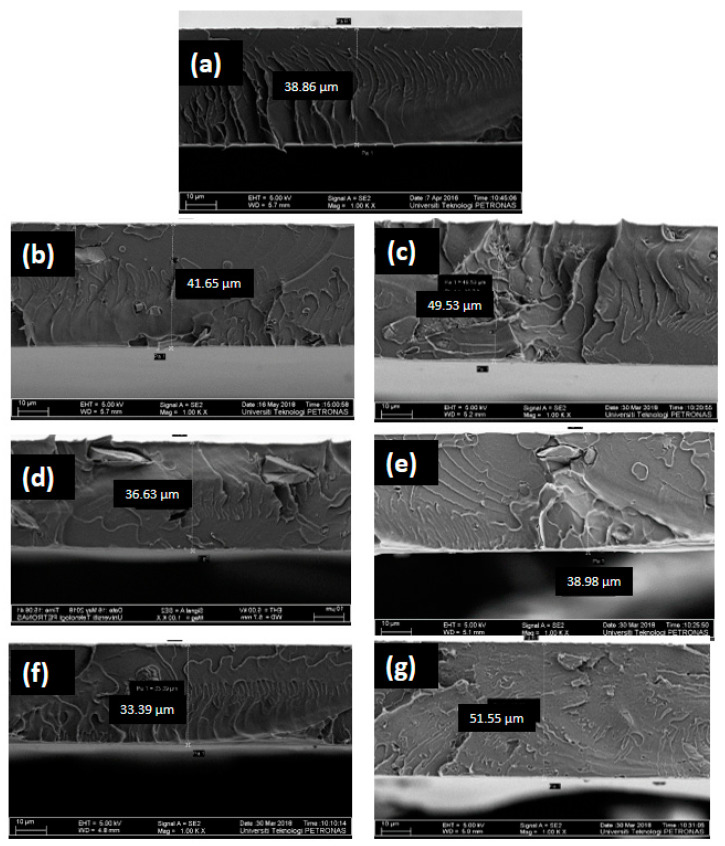
FESEM images of (**a**) pure PC, (**b**) 1 PC-Sil, (**c**) 1 PC-Sil-IL, (**d**) 2 PC-Sil, (**e**) 2 PC-Sil-IL, (**f**) 3 PC-Sil, (**g**) 3 PC-Sil-IL.

Figure 3 shows the cross-section morphology of all prepared membranes. All membranes appear as dense membranes which is expected from the dry phase inversion technique used for the membrane preparation [7]. The incorporation of pure Sil particles and Sil-IL particles induced some changes in the morphology of the membranes. PC-Sil and PC-Sil-IL MMMs were observed to have heterogeneous structures where irregularly shaped Sil particles and Sil-IL particles, respectively, were present in the PC matrix (Figure 2). It is noted that no particular difference was observed between PC-Sil MMM and PC-Sil-IL MMM.

**Figure 4:**
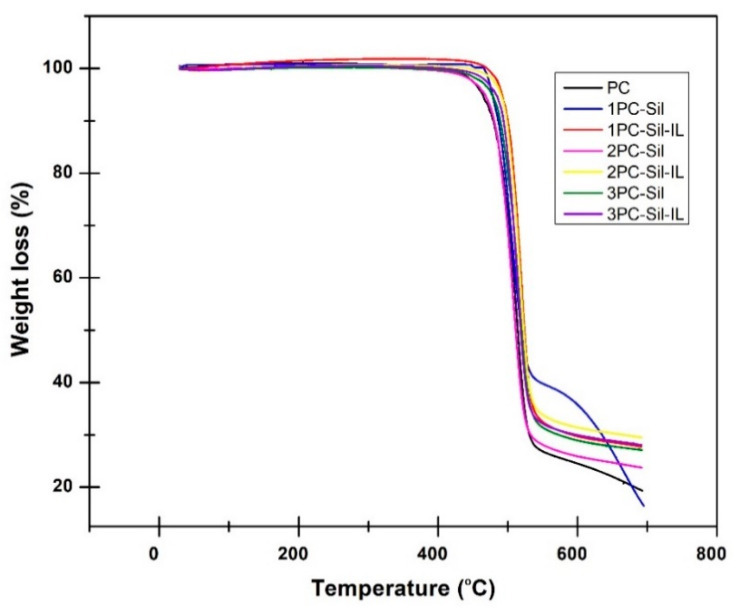
Thermal stability of the developed membranes.

The thermal stability of the prepared membrane was examined using TGA and the result is presented in Figure 4. It can be observed that the pure PC membrane experienced steep initial weight loss at 450 °C. The thermal degradation temperature was observed to range from 450 to 550 °C, which is attributed to the PC polymer decomposition. Meanwhile, PC-Sil MMMs and PC-Sil-IL MMMs displayed initial thermal degradation at 450 °C and the weight loss continued until 550 °C. The weight loss was due to the thermal degradation and decomposition of functional groups in Sil particles [7]. It can be concluded that all fabricated MMMs were thermally stable until 450 °C before the functional groups started to decompose.

The DSC analysis was conducted to investigate the effects of the incorporated Sil fillers and Sil-IL fillers on the glass transition temperatures (T_g_) of the PC membrane and the results are presented in Table 3. Upon incorporation of 1 wt% Sil, the T_g_ of the membrane was reduced. This indicates that the polymer matrix became more flexible. However, increasing the Sil loading (2 and 3 wt%), increased the T_g_ of the prepared MMM, albeit lower than neat PC membrane. This could be due to the larger amount of Sil that interacted with the PC matrix, thus increasing the rigidity of the membrane. Moreover, incorporating 1 wt% Sil-IL reduced the T_g_ of the membrane. It can be postulated that IL increased the mobility in the polymer chain and acted as a plasticizer in the PC matrix. The reduction in T_g_ was similarly reported in a study where the addition of low molecular weight additives decreased the T_g_ value of the polymer membrane [31]. Low molecular weight additives like [EMIM][TF_2_N] reduced the membrane rigidity and brittleness, resulting in the softening of the polymer matrix [32]. However, with increasing loading of Sil-IL (2 and 3 wt%), the T_g_ remained almost the same as the PC-Sil membrane of the same loading. It is postulated that as the fillers loading increased, the interaction of Sil particles increased, thus increasing the membrane rigidity, disregarding the presence of IL.

The intersegmental distance or d-spacing of the resulting membranes is summarized in Table 3. The *d*-spacing for the MMMs decreased gradually with increasing filler loading. The decrease in the *d*-spacing could also signify the decrement in free fractional volume (FFV) in the membrane caused by chain rigidification after the filler was incorporated, as suggested, by the increment in the membranes’ T_g_. The decrease in *d*-spacing for Sil-IL membranes was also more prominent than for the Sil membrane counterpart. This is likely due to interaction between the IL with the polymer matrix that reduced the intersegmental spacing of the polymer chain.

### 3.3. Gas Permeation Test

#### 3.3.1. Effect of Sil-IL and Its Loading

**Figure 5:**
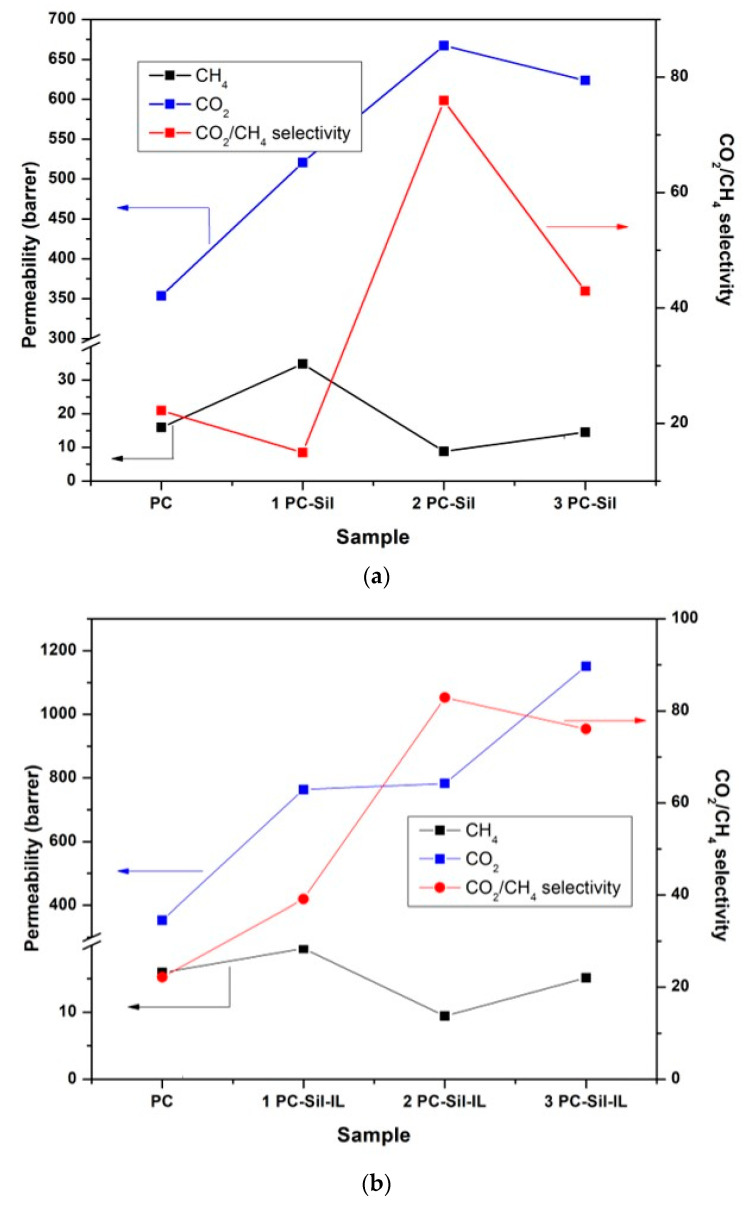
Separation performance of the prepared membranes in Barrer for (**a**) PC-Sil MMM, (**b**) PC-Sil-IL MMM at 2 bar feed pressure.

Figure 5 shows the gas separation performance of PC, PC-Sil MMMs, and PC-Sil-IL MMMs at a feed pressure of 2 bar. For CO_2_ permeability, the pure PC membrane showed initial permeability at 350 Barrer. With the incorporation of pure Sil, the permeation increased by 48%, 91%, and 78% for 1 PC-Sil, 2 PC-Sil, and 3 PC-Sil, respectively, compared to pure PC membrane (Figure 5a). It is expected that as Sil particle loading increased, more free volumes were created (Figure 1), resulting in the rise in the CO_2_ permeability [33]. Interestingly, 1 PC-Sil IL shows an increment in CO_2_ permeability by 87%, 2 PC-Sil-IL by 28%, and 3 PC-Sil-IL by 77% (Figure 5b) compared to 1 PC-Sil, 2 PC-Sil, and 3 PC-Sil, respectively. The increase in CO_2_ permeability is postulated to be due to the presence of flouroalkyl chains from [EMIM][TF_2_N] that provide high CO_2_ affinity. In previous studies [33,34,35], it was reported that CO_2_ is more soluble in [EMIM][TF_2_N] compared to CH_4_ (CO_2_/CH_4_ solubility selectivity of 11.2). Therefore, in can be presumed that modifying the silica using the IL provides improvements in the CO_2_ permeability, specifically the solubility.

It should be noted that gas permeation across a membrane is dependent on two factors: solubility and diffusivity. As the *d*-spacing decreased when increasing Sil and Sil-IL loading (Table 3), this signifies that the diffusivity coefficient was reduced for the prepared MMMs. Therefore, the increases in the CO_2_ permeability were dominated by the solubility coefficient, with Sil-IL being shown to have a higher solubility coefficient compared to Sil as filler.

PC membrane shows a comparable CO_2_/CH_4_ selectivity value of 18, slightly higher compared to the literature [7]. Overall, all MMMs show higher CO_2_/CH_4_ selectivity than that of the pure PC membrane except for 1 PC-Sil. The decrease in the *d*-spacing of the prepared MMMs (Table 3) would provide minimal diffusion sites for gas to permeate. As CH_4_ has a kinetic diameter of 3.8 Å, the gas diffusion across the membrane was restricted and resulted in lower permeability. With higher CO_2_ affinity and restricted CH_4_ diffusion across the membrane, the incorporated filler, especially for Sil-IL MMMs, resulted in superior CO_2_/CH_4_ selectivity compared to neat PC membrane.

#### 3.3.2. Effect of Pressure

**Figure 6:**
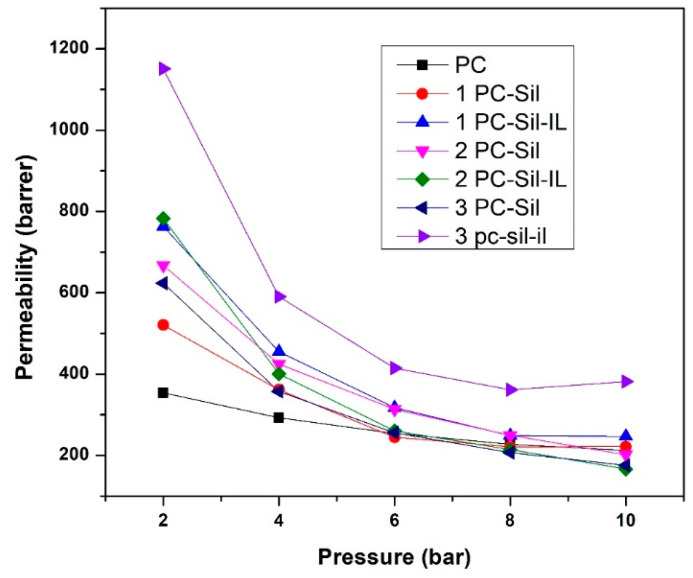
CO_2_ permeability of fabricated PC, PC-Sil MMMs, and PC-Sil-IL MMMs at pressure ranging from 2 to 10 bar.

The effect of feed pressure (2–10 bar) on the prepared membrane performance is presented in Figure 6. In general, all developed MMMs have higher CO_2_ permeability at low pressure compared to pure PC. At both the lowest and the highest pressure, 3 PC-Sil-IL shows the highest permeability at 1151 and 420 Barrer, respectively. As the pressure increases, the CO_2_ permeability of all membranes shows a decreasing trend, following the dual-mode sorption. At low pressure, gas sorption increases progressively as the gas fills up both equilibrium and excess free volumes. As pressure increases, the total sorption of polymer increases linearly after Langmuir sorption sites are saturated, decreasing the permeability of the gas [36].

**Figure 7:**
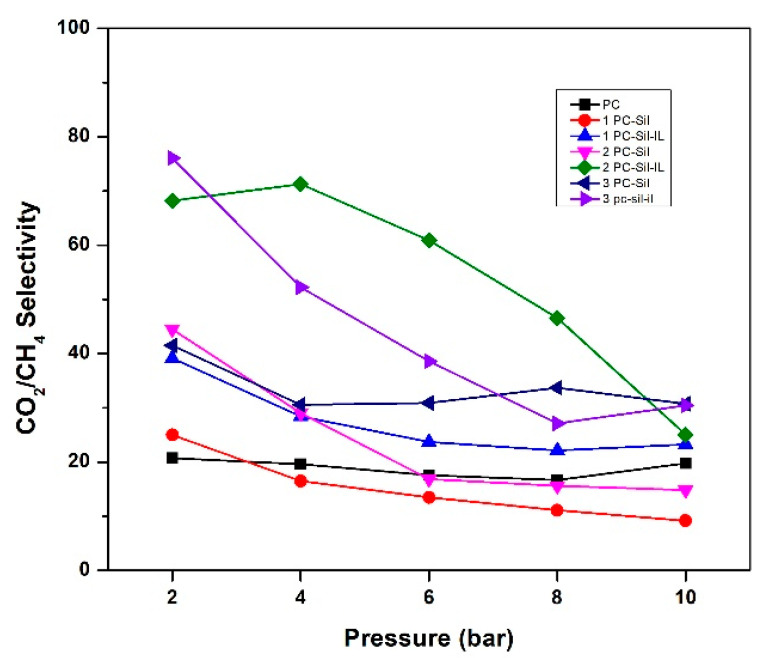
CO_2_/CH_4_ selectivity of fabricated PC, PC-Sil MMMs, and PC-Sil-IL MMMs at pressure ranging from 2 to 10 bar.

Furthermore, all MMMs except 3 PC-Sil and 1 PC-Sil-IL show a downward trend with increasing pressure for CO_2_/CH_4_ selectivity, as shown in Figure 7. The trend is attributed to the sudden decrease in CO_2_ permeability as CO_2_ has a higher solubility effect compared to CH_4._ Thus, when the pressure increases, the free volumes in the membrane matrix become saturated, thus decreasing both the CO_2_ permeability and the CO_2_/CH_4_ selectivity.

#### 3.3.3. Comparison with Robeson Upper Bound

**Figure 8:**
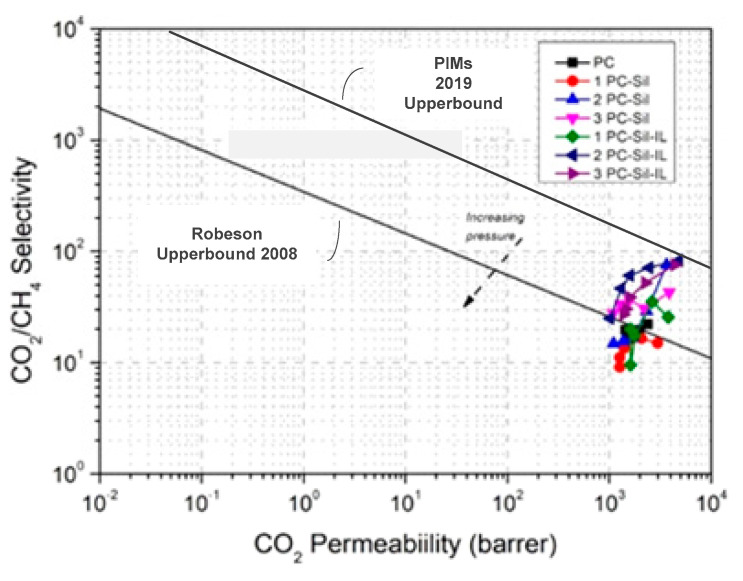
Comparison of separation performance for fabricated PC, PC-Sil, PC-Sil-IL MMMs with 2008 Robeson upper bound and PIMs 2019 redefined upper bound [37,38].

The gas permeation performances of all MMMs are plotted on the 2008 Robeson upper bound [37] and PIMs 2019 redefined upper bound [37], as shown in Figure 8. The CO_2_ permeability in Barrer is calculated using Equation (2) with the average thickness of the membranes obtained from FESEM images (Figure 3). It can be observed that almost all MMM performances exceed the Robeson 2008 upper bound for CO_2_ separation, with the exception of 1 PC-Sil, 2 PC-Sil (low loading), and 1 Pc-Sil-IL (low loading), albeit still lower than the PIMs 2019 redefined upper bound. Meanwhile, from the plot, 2 PC-Sil-IL and 3 PC-Sil-IL show promising results at low pressure. However, due to the dual-mode sorption, the performance is observed to fall towards the line. This suggests that the application of [EMIM][TF_2_N]-modified Sil on the performance of polymeric membranes shows promising results for CO_2_ separation.

## 4. Conclusions

The effects of filler modification and filler loading on PC membranes were investigated and discussed. Sil fillers were successfully modified via surface modification as indicated by the functional group in FTIR and fluorine elements from [EMIM][TF_2_N] in XPS. The incorporation of Sil and Sil-IL formed heterogenous structures on the PC membrane. Moreover, all MMMs showed good thermal stability until 450 °C, before the functional groups started to degrade. Incorporating Sil and IL-modified Sil particles into the polymer membrane reduced the T_g_ of the PC membrane as the Sil fillers increased the flexibility of the polymer matrices. From the permeation test, incorporating IL-modified Sil increased the CO_2_ permeability and CO_2_/CH_4_ selectivity compared to the pure PC membrane at low pressure (6 bar and below). 3 PC-Sil-IL showed the most promising result with an increment of four times in CO_2_/CH_4_ selectivity. All fabricated Sil-IL MMMs were able to surpass the 2008 Robeson upper bound except for 1 PC-Sil, 2 PC-SIL, 1PC-Sil-IL, and 2PC-Sil-IL at low loading but they still performed below the PIMs 2019 redefined upper bound, indicating promising applications in CO_2_ separation. However, further investigation regarding the Sil-IL MMMs at high pressure should be considered for applications in natural gas processing.

## Figures and Tables

**Table 1:** Composition of PC, DCM, and inorganic fillers for developed MMMs.

Sample Name	PC (wt%)	Solvent (wt%)	Sil (wt% of Total Solid)	Sil-IL (wt% of Total Solid)
PC	20	80	-	-
1 PC-Sil	20	80	1	-
2 PC-Sil	20	80	2	-
3 PC-Sil	20	80	3	-
1 PC-Sil-IL	20	80	-	1
2 PC-Sil-IL	20	80	-	2
3 PC-Sil-IL	20	80	-	3

**Table 2:** Elemental composition of Sil and Sil-IL particles.

Sample	Element (wt%)				
	Si	N	O	F	C
Pure Si	34.03	-	65.97	-	-
Si-IL	42.63	1.16	50.47	1.2	4.54

**Table 3:** Glass transition temperature (T_g_) and *d*-spacing of the developed membranes.

Sample	T_g_ (°C)	d (Å)
Pure PC	144.4	4.91
1 PC-Sil	142.1	4.93
2 PC-Sil	143.9	4.83
3 PC-Sil	143.3	4.68
1 PC-Sil-IL	141.5	4.89
2 PC-Sil-IL	143.8	4.79
3 PC-Sil-IL	143.3	4.66
